# Circadian blood pressure variability in type 1 diabetes subjects and their nondiabetic siblings - influence of erythrocyte electron transfer

**DOI:** 10.1186/1475-2840-9-61

**Published:** 2010-10-05

**Authors:** Elena Matteucci, Cristina Consani, Maria Chiara Masoni, Ottavio Giampietro

**Affiliations:** 1 Department of Internal Medicine, University of Pisa, via Roma 67, 56126 Pisa, Italy

## Abstract

**Background:**

Normotensive non-diabetic relatives of type 1 diabetes (T1D) patients have an abnormal blood pressure response to exercise testing that is associated with indices of metabolic syndrome and increased oxidative stress. The primary aim of this study was to investigate the circadian variability of blood pressure and the ambulatory arterial stiffness index (AASI) in healthy siblings of T1D patients vs healthy control subjects who had no first-degree relative with T1D. Secondary aims of the study were to explore the influence of both cardiovascular autonomic function and erythrocyte electron transfer activity as oxidative marker on the ambulatory blood pressure profile.

**Methods:**

Twenty-four hour ambulatory blood pressure monitoring (ABPM) was undertaken in 25 controls, 20 T1D patients and 20 siblings. In addition to laboratory examination (including homeostasis model assessment of insulin sensitivity) and clinical testing of autonomic function, we measured the rate of oxidant-induced erythrocyte electron transfer to extracellular ferricyanide (RBC vfcy).

**Results:**

Systolic blood pressure (SBP) midline-estimating statistic of rhythm and pulse pressure were higher in T1D patients and correlated positively with diabetes duration and RBC vfcy; autonomic dysfunction was associated with diastolic BP ecphasia and increased AASI. Siblings had higher BMI, lower insulin sensitivity, larger SBP amplitude, and higher AASI than controls. Daytime SBP was positively, independently associated with BMI and RBC vfcy. Among non-diabetic people, there was a significant correlation between AASI and fasting plasma glucose.

**Conclusions:**

Siblings of T1D patients exhibited a cluster of sub-clinical metabolic abnormalities associated with consensual perturbations in BP variability. Moreover, our findings support, in a clinical setting, the proposed role of transplasma membrane electron transport systems in vascular pathobiology.

## Background

So far, familial predisposition to arterial hypertension in type 1 diabetes (T1D) families has been related to the development of diabetic nephropathy and systemic hypertension in the proband [1]. We have previously shown that normotensive non-diabetic siblings of T1D patients have abnormal blood pressure response to exercise testing that is associated with indices of metabolic syndrome and increased oxidative stress [2]. The primary aim of this study was to investigate the circadian variability of blood pressure and the ambulatory arterial stiffness index in healthy siblings of T1D patients vs healthy control subjects who had no first-degree relative with T1D. Secondary aims of the study were to explore the influence of cardiovascular autonomic function and erythrocyte electron transfer activity on the ambulatory blood pressure profile. Human erythrocytes possess a transplasma ferricyanide reductase activity (measured as the erythrocyte velocity of ferricyanide reduction, RBC vfcy) that transfers reducing equivalents from intracellular reductants to extracellular oxidants [3] and belongs to the ubiquitous transplasma membrane electron transport (TPMET) systems. TMET activities have been related to the regulation of vital cellular processes and to the pathogenesis of various human disorders [4] and exist also in endothelial cells where they have been suggested to regulate redox status and possibly atherogenesis through regulation of haeme oxygenase-1 expression [5].

## Methods

### Participants

We studied the following groups of subjects:

1) 20 T1D patients (12 women) with duration of disease ranging from 1 to 47 years (23 ± 15 years). The group included 8 patients with retinopathy (background or proliferative, determined by fluorescein angiography following fundus examination) and 1 patient with nephropathy (persistent macroalbuminuria). None received medical treatment except insulin (0.6 ± 0.2 IU/kg) and possibly antihypertensive drugs (the patient with nephropathy was taking angiotensin converting enzyme inhibitor and calcium antagonist);

2) 20 normotensive non-diabetic siblings (12 women) of T1D patients. None had clinical evidence of illness or was taking any drugs;

3) 25 healthy subjects (16 women) were recruited from the local community to achieve a similar distribution of age and sex to the families. They had no family history of T1D, were taking no drugs, and had no clinical signs or symptoms of illness.

All subjects gave informed consent and the ethical committee of the hospital approved the study proposal. The medical examination included standardised family and personal medical history, physical examination, blood chemistry analysis, tests for cardiovascular autonomic functions, resting ECG, and 24-h ambulatory blood pressure monitoring (ABPM).

### Data collection

The family and personal medical history and the findings of the clinical examination were recorded (body mass index, blood pressure, and cigarette smoking included). The body mass index (BMI) was calculated as body weight/height^2 ^(in kg/m^2^). After 10 minutes rest, sitting systolic and diastolic blood pressure were measured twice. The mean was reported as office SBP and DBP. Individuals not receiving any antihypertensive drug therapy with resting (sitting) SBP/DBP less than 140/90 mmHg were placed in normal blood pressure category.

### Tests of autonomic function

Autonomic function was assessed using four standardised autonomic function tests according to Ewing et al. [6]: deep breathing, lying to standing, Valsalva manoeuvre, and orthostatic hypotension. Heart rate variability (HRV) was analysed using NeuroTester device (Me.Te.Da. srl, San Benedetto del Tronto, Italy) and evaluated using age-related reference values [7]. Expiration/inspiration ratio at deep breathing (E:I), maximum/minimum ratio after change in position (30:15), and Valsalva ratio were scored as 0 (normal), 1 (borderline), or 2 (abnormal). Orthostatic fall in SBP (Standing ΔSBP) was defined abnormal if > 20 mmHg. Patients with at least one abnormal or two borderline cardiovascular tests (score ≥2) were considered to have autonomic neuropathy [8].

### Ambulatory blood pressure monitoring

The 24-h ABPM was performed using an automatic oscillometric device (Takeda TM-2430) and data were analysed using SIGMA 2000 software. Patients should keep their habitual routine and present a report with the activities done. SBP, DBP and heart rate (HR) were recorded every 15 minutes throughout the day and every 30 minutes at night. SBP, DBP, and HR measurements were averaged for the day and the night spans according to the patients' reported time of waking up and going to bed. The pulse pressure (PP) was calculated as the difference between the SBP and the DBP. The normal dip was defined as a 10% or greater reduction in BP during sleep compared with the awake period. The circadian characteristics of BP were also estimated: MESOR (midline-estimating statistic of rhythm), amplitude (half of difference between highest and lowest value of curve), and acrophase (timing of highest value in curve). Ambulatory arterial stiffness index (AASI) was computed from 24-h recordings of each participant as 1 minus the regression slope of DBP on SBP [9].

### Laboratory methods

Fasting venous blood samples and morning urine samples were obtained on the same day just before starting ABPM. Measurements were performed in freshly obtained material immediately after withdrawal. Laboratory evaluation included fasting plasma glucose (FPG), plasma creatinine and uric acid, HbA1c, blood cell count, lipids (total, HDL, LDL cholesterol, and triglycerides), bilirubin, liver enzymes, urinary glucose, albumin and creatinine excretion. Urinary albumin-to-creatinine ratio (ACR) was calculated by dividing the urinary albumin concentration in μg by the urinary creatinine mg. Concentrations of fasting plasma insulin (FPI) were measured by commercial radioimmunoassay (Medgenix Diagnostics, Fleurus, Belgium). Homeostasis model assessment of insulin sensitivity (HOMA-IS) was calculated as the product of the FPI and FPG divided by 22.5.

To measure RBC vfcy, 4 ml venous blood were drawn into heparin tubes and immediately centrifuged at 54 g for 9 min at 20°C to remove platelet-rich plasma. After packed erythrocytes were washed three times in 10 volumes of phosphate-buffered saline, 0.5 ml of the packed cells were diluted to 5 ml with phosphate-buffered saline containing l mmol/l ferricyanide and incubated under magnetic stirring in a water bath at 37°C. At 5, 10, 20 and 40 minutes 0.3 ml of cell suspension were removed and centrifuged at 3110 g for 4 min at 4°C. An aliquot of 0.05 ml of the supernatant was sampled, and the ferrocyanide content was measured spectrophotometrically (from its absorbance at 535 nm with 4,7-diphenyl-l,10-phenanthroline sulfonated as the color-developing agent against a 0 time blank). The initial rate of ferrocyanide generation was expressed relative to the packed cell volume as micromoles per ml packed RBCs per hour [3].

### Statistical analyses

Analysis was performed using Aabel 3 (Gigawiz, Oklahoma City, Oklahoma, USA). Results are given as mean ± SD or median with range (Standing ΔSBP, triglycerides, and ACR). Statistical comparison was by chi-square test, the Student's unpaired *t *test or the Mann-Whitney U-test with Bonferroni correction for multiple hypothesis testing. The cut-off level for statistical significance was set at p < 0.05. Pearson's correlation coefficient or Spearman's rho was computed to determine the correlation between variables. Multivariate regression analysis was used to determine independent predictors of the variables of interest; candidate predictors selected in the multivariate model were the variables significantly associated by simple univariate correlation analysis.

## Results

The clinical characteristics of the study groups are reported in Table 1. T1D patients had higher office SBP (duplicate measurements) than control subjects, but still in the normal range. Their siblings showed slightly higher BMI than control subjects, but similar office BP levels.

**Table 1:** Clinical characteristics of the study participants

Characteristic	Controls	T1D patients	Siblings
Age (years)	46 ± 12	46 ± 10	45 ± 10
BMI (kg/m^2^)	24 ± 3	25 ± 4	26 ± 5*
Office SBP (mmHg)	117 ± 9	126 ± 14•	118 ± 21
Office DBP (mmHg)	79 ± 4	79 ± 9	82 ± 12
Never smoker/smoker/ex	14/6/5	11/4/5	13/2/5
Deep Breathing (E:I)	1.36 ± 0.14	1.33 ± 0.12	1.36 ± 0.17
Lying to Standing (30:15)	1.42 ± 0.39	1.20 ± 0.14*	1.45 ± 0.28‡
Valsalva ratio	2.07 ± 0.70	1.68 ± 0.33*	1.95 ± 0.46†
Standing ΔSBP	[-5, -20 +10]	[-10, -45 +10]	[-8, -30 +10]

Data are mean ± SD or [median, range]. *p < 0.05, •p < 0.01, ¶p < 0.001 comparing T1D or their siblings vs. normal control subjects; †p < 0.05, ‡p < 0.01, #p < 0.001 comparing siblings vs. T1D.

BMI, body mass index; SBP, systolic blood pressure; DBP, diastolic blood pressure; Standing ΔSBP, orthostatic fall in SBP.

While HR analysis under paced breathing did not evidence any significant difference among groups, HRV in response to standing and Valsalva manoeuvre was abnormal in T1D patients. Five of them had an autonomic neuropathy score ≥2.

From the biochemical viewpoint (Table 2), T1D patients differed from both the control subjects and their own siblings in the higher FPG and HbA1c (as expected) as well as in the lower plasma levels of triglycerides; they also had lower plasma LDL cholesterol than siblings. Siblings differed from the control group in the higher FPG but lower HbA1c. The range of FPG was from 4.0 to 5.8 mmol/l (FPI from 2.2 to 28.7 μU/ml) in control subjects and from 4.4 to 6.1 (FPI from 6.3 to 42.3) in siblings. HOMA-IS was significantly lower in siblings than the control group (0.48 ± 0.20 vs 0.70 ± 0.43, p < 0.05).

**Table 2:** Biochemical characteristics of the study participants

Characteristic	Controls	T1D patients	Siblings
FPG (mmol/l)	4.9 ± 0.4	10.5 ± 3.6¶	5.2 ± 0.5*#
HbA1c (%)	5.3 ± 0.3	8.6 ± 1.4¶	5.1 ± 0.3*#
Total cholesterol (mmol/l)	5.2 ± 0.7	4.9 ± 0.9	5.3 ± 1.0
HDL cholesterol (mmol/l)	1.5 ± 0.4	1.6 ± 0.3	1.4 ± 0.3
LDL cholesterol (mmol/l)	2.9 ± 0.6	2.8 ± 0.7	3.2 ± 0.9†
Triglycerides (mmol/l)	[1.0, 0.4-1.7]	[0.7, 0.4-1.2]°	[0.8, 0.5-1.7]†
Plasma creatinine (μmol/l)	84 ± 13	93 ± 10*	87 ± 12
ACR (μg/mg)	[3.2, 0-18]	[2.5, 0-1395]	[2.5, 0-12]
RBC vfcy (mmol/ml h)	10.1 ± 2.5	9.6 ± 2.6	10.4 ± 2.9

Data are mean ± SD or [median, range]. *p < 0.05, °p < 0.01, ¶p < 0.001 comparing T1D or their siblings vs. normal control subjects; †p < 0.05, ‡p < 0.01, #p < 0.001 comparing siblings vs. T1D.

FPG, fasting plasma glucose; HDL, high density lipoprotein; LDL, low density lipoprotein; ACR, albumin-to-creatinine ratio; RBC vfcy, erythrocyte velocity of ferricyanide reduction.

**Figure 1:**
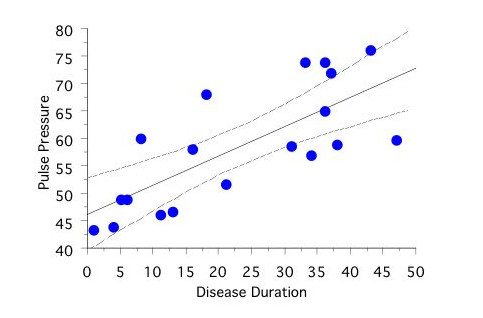
**Relationship between 24-h pulse pressure (mmHg) and duration of type 1 diabetes (years)**.

**Figure 2:**
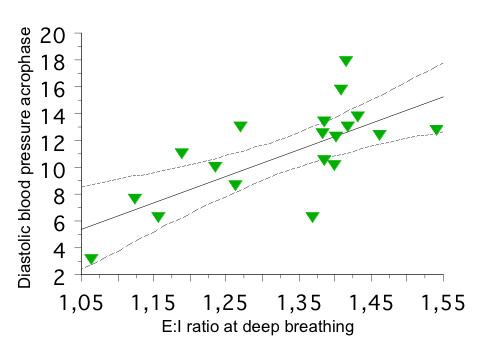
**Linear regression plot of diastolic blood pressure (DBP) acrophase in type 1 diabetic patients vs. heart rate variability in response to deep breathing, expressed as expiration/inspiration ratio (E:I)**.

ABPM confirmed the 24-h SBP, 24-h PP, nighttime SBP and SBP MESOR to be significantly higher in T1D patients (one T1D patient with recording duration of 20 hours, who declined further attempts, was excluded from the correlation analysis) than in control subjects and siblings (Table 3). In bivariate correlation analysis, 24-h PP correlated positively and significantly with the duration of diabetes (r = 0.75, p < 0.001; Figure 1), HbA1c (r = 0.64, p < 0.01), RBC vfcy (r = 0.59, p < 0.01), and autonomic neuropathy score (r = 0.46, p < 0.05). In multivariate analyses, duration of diabetes (β coefficient = 0.45, p < 0.001) and RBC vfcy (β = 1.70, p = 0.01) resulted to be independently related to PP (r = 0.84, p < 0.001). Moreover, in T1D patients DBP acrophase occurred earlier (despite the same time of awakening and in the absence of an oddly timed pattern of HR) and was positively associated (r = 0.70) with HRV in response to deep breathing after controlling for age and diabetes duration (β = 19.52, p = 0.01, Figure 2).

**Table 3:** Measures of ambulatory blood pressure monitoring

Characteristic	Controls	T1D patients	Siblings
24-h SBP (mmHg)	121 ± 7	135 ± 12¶	127 ± 13†
24-h DBP (mmHg)	74 ± 4	77 ± 5	78 ± 8
24-h PP (mmHg)	46 ± 4	58 ± 11¶	49 ± 8‡
AASI (units)	0.47 ± 0.13	0.62 ± 0.15#	0.56 ± 0.13*
Daytime SBP (mmHg)	125 ± 7	139 ± 12¶	132 ± 14*
Daytime DBP (mmHg)	77 ± 4	79 ± 7	81 ± 8*
Nighttime SBP (mmHg)	107 ± 10	122 ± 17°	108 ± 15†
Nighttime DBP (mmHg)	65 ± 8	68 ± 7	66 ± 8

SBP Mesor (mmHg)	121 ± 6	135 ± 12¶	127 ± 13†
SBP Amplitude (mmHg)	10 ± 5	12 ± 5	14 ± 6°
SBP Acrophase (h)	14 ± 3	14 ± 5	14 ± 2
DBP Mesor (mmHg)	75 ± 4	77 ± 5	78 ± 8
DBP Amplitude (mmHg)	7 ± 3	6 ± 4	8 ± 4
DBP Acrophase (h)	14 ± 3	11 ± 3°	14 ± 2#

Data are mean ± SD. *p < 0.05, °p < 0.01, ¶p < 0.001 comparing T1D or their siblings vs. normal control subjects; †p < 0.05, ‡p < 0.01, #p < 0.001 comparing siblings vs. T1D.

PP, pulse pressure; AASI, ambulatory arterial stiffness index.

**Figure 3:**
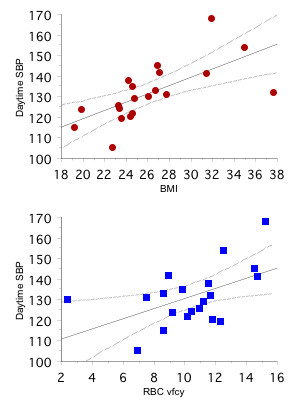
**Linear regression plots of daytime systolic blood pressure (SBP, mmHg) in siblings vs. BMI (upper panel) and erythrocyte transplasma membrane electron transport, RBC vfcy as micromoles of ferrocyanide generated per milliliter packed RBCs per h (lower panel)**.

Unlike office duplicate BP measurements, ABPM records of siblings revealed the daytime SBP, daytime DBP, and SBP amplitude to be higher than controls' values. Among non-diabetic people in the study, daytime SBP was associated positively with BMI (r = 0.55, p < 0.001), HbA1c (r = 0.36, p = 0.01), RBC vfcy (r = 0.34, p < 0.05; Figure 3) and negatively with HOMA-IS (r = -0.39, p < 0.01). On multivariate regression model analysis, the relationship for BMI (β = 1.48, p < 0.001) and RBC vfcy (β = 1.04, p < 0.01) with daytime SBP remained (r = 0.61, p < 0.001).

Five control subjects, 6 T1D patients, and 1 sibling had a non-dipping pattern of SBP on the ABPM; 4 control subjects, 7 T1D patients, and 1 sibling had a non-dipping pattern of DBP. The total score for autonomic neuropathy (the sum of the four test scores) accounts for the major part of the non-dipping systolic (r = -0.58, p < 0.01) and diastolic (r = -0.70, p < 0.001) profile in the T1D patients.

AASI was higher in members of T1D families, probands and siblings, than in control subjects. In T1D patients it was associated negatively with E:I ratio at deep breathing (r = -0.67, p = 0.001) and positively with age (r = 0.63, p < 0.01), duration of the disease (r = 0.53, p < 0.05), and HbA1c (r = 0.48, p < 0.05). On multivariate regression HRV during deep breathing remained the independent predictor of AASI (β = -0.69, p < 0.01) even when 24-h SBP was included in the model. In the non-diabetic study groups there was a significant positive relationship between AASI and FPG (r = 0.29, p < 0.05).

## Discussion

Ambulatory BP, particularly the circadian variability of BP, may be a better predictor of cardiovascular disease than office BP in the general population [10]. Abnormal circadian BP patterns have been described in high-risk T1D patients with diabetic nephropathy and/or autonomic neuropathy [11,12]. Our T1D patients, 95% of whom were normoalbuminuric, had higher 24-h PP (due to higher MESOR, daytime and nighttime SBP) whose variance was mainly accounted for disease duration, thus confirming recent observations [12]. Philips el al. measured SBP and DBP continuously with a Finapres (FINger Arterial PRESsure) device and found a progressive PP increase in T1D patients according to disease duration, especially after 20 years of diabetes, in an age range (< 50 years) where such a PP rise was not observed in non-diabetic individuals. In our T1D patients, cardiovascular autonomic neuropathy was independently correlated with their higher AASI. Normoalbuminuric T1D patients have been found to have increases in arterial stiffness, which, in turn, were associated with, and could cause, increased systolic and pulse pressure [13]. One of the main mechanisms thought to be involved is the formation of advanced glycation end products [14]. However, BP alterations are early occurrences in T1D [15] and acute hyperglycaemia alone (120 min hyperglycaemic clamp) is sufficient to increase the stiffness of intermediate-sized arteries and resistance arteries in young T1D people without diabetic complications as well as in healthy subjects in a rather similar manner [16]. Indices of cardiovascular autonomic neuropathy also deteriorate with diabetes duration and shifts of the sympathovagal balance might in part account for vascular dysfunction [11], as our data suggest.

The acrophase of DBP, i.e. the time of the maximum of the DBP, registered in T1D probands was 3 hours earlier than normal and DBP ecphasia (altered circadian timing) was more pronounced in patients with lower HR variability during deep breathing. This is a novel finding in type 1 diabetes, which is consistent with previous observations in patients with type 2 diabetes [17]. Changes in the time structures of melatonin and BP have been described in diabetes and associated with autonomic nervous dysfunction [18]. Recent results prove that streptozotocin-induced T1D increases the plasma melatonin level and confirm a melatonin-insulin antagonism [19]. In T1D, despite sophisticated regimens of subcutaneous insulin substitution, the elevated levels of circulating insulin could impact on the circadian regulation of BP by melatonin.

Among previous investigations focused on the relationship between familial predisposition to hypertension and diabetic nephropathy, only one monitored 24-h ambulatory BP in adult T1D relatives (parents aged about 67 years) [1]. The present study is first to evaluate 24-h ambulatory BP in healthy siblings of similar adult age as the (mostly uncomplicated) probands. Siblings differed from control subjects in the following respects: higher BMI and FPG, lower HbA1c and HOMA-IS, abnormal diurnal profiles of SBP at ABPM, and abnormal dynamic relationship between SBP and DBP over 24 h as described by AASI.

Originally, the presence of insulin resistance was highlighted in family members of T1D patients with microalbuminuria [20]. Currently, the accelerator hypothesis proposes that type 1 and type 2 diabetes are both driven by insulin resistance [21]. In the course of our studies on non-diabetic first-degree T1D relatives [2,22], we ourselves have repeatedly documented the tendency towards a similar metabolic derangement. In the present study, siblings had FPG slightly higher than control subjects yet lower HbA1c levels. According to the 2003 ADA definition, 6 siblings (vs 2 control subjects) had impaired fasting glucose (IFG). Having FPG < 7.0 mmol/l and HbA1c < 6.0%, our siblings presumably did not require an oral glucose tolerance test to exclude diabetes [23]. Five-year follow-up data from the Inter99 study evidenced that the proportion of individuals with a family history of diabetes was highest in the groups who later developed IFG and IFG/IGT (impaired glucose tolerance). The pre-diabetic state, IFG, was characterised by a baseline stationary reduced insulin secretion in combination with a progressive decline in HOMA-IS, assumed to reflect hepatic insulin sensitivity [24]. The combination of higher mean FPG, lower HOMA-IS and lower HbA1c levels suggested that siblings with isolated IFG (thus impaired basal glucose clearance rate) may also have lower postprandial glycaemia values (due to enhanced insulin-stimulated muscle glucose disposal) [25]. Recently, 7-day ABPM uncovered that prediabetics had higher incidence of high mean BP, excessive PP and/or circadian hyper-amplitude-tension (a circadian BP amplitude above the upper 95% prediction limit of healthy peers matched by gender and age) [26]. Similarly, our siblings reached higher values of daytime SBP and SBP amplitude than controls.

A circadian hyper-amplitude-tension (based on a chronobiological classification) has been associated with an increased risk of cardiovascular diseases such as stroke, coronary artery disease, nephropathy, and retinopathy [27]. Longitudinal studies in animal models of hypertension have reported that an increase in the circadian amplitude of BP precedes a rise in the circadian BP average or MESOR hypertension [28]. Thus, in accordance with scientific evidence, these siblings seem to be in a prediabetes and prehypertension phase. In nondiabetic, normotensive participants in the San Antonio Heart Study, subjects with prehypertension were at increased risk of diabetes and much of this risk was accounted for disorders related to the insulin resistance syndrome [29]. Plasma glucose level is likely to be a continuous cardiovascular risk factor even within a range that is below the diabetic threshold. In hypertensive patients with type 2 diabetes, nocturnal systolic blood pressure and hyperglycemia were co-factors for an increased prevalence of left ventricular hypertrophy [30]. Nevertheless, in our multivariate model, siblings' mean daytime SBP was independently related only to BMI and RBC vfcy. Indeed, even a mild increase of BMI increased the risk of prehypertension [31], as found here. Familiarity for type 2 diabetes showed some influence on autonomic nervous system. A global reduction and alteration of circadian rhythm of autonomic activity (evaluated by HRV with 24-hours ECG Holter registration) were present in nondiabetic offspring of type 2 diabetic patients [32]. In our study, however, tests of autonomic function were normal in siblings.

Present true novelty lies in that the daytime SBP, as well as PP, is partly explained by RBC vfcy. All eukaryotic cells possess TMET systems that transfer electrons across the plasma membrane; the reduction of extracellular oxidants takes place at expense of intracellular reductants (NADH, NADPH, ascorbate, glutathione, etc.). TMET capacity has been related to various cellular functions: growth, proliferation, differentiation, apoptosis, bioenergetics, cell signal transduction, antioxidation, and iron/copper metabolism [4]. Consequently, TPMET dysfunction has been implied in various human pathologies, diabetes included [3], even though the molecular structures responsible for the trans-plasma membrane transfer remain incompletely identified. Suggested mechanisms of this transfer are probably twofold: 1) enzyme-mediated electron transfer through the redox centers of plasma membrane oxidoreductases, and 2) transmembrane metabolite shuttling/cycling (particularly ascorbate/dehydroascorbate cycling by plasma membrane conduits and/or exocytosis) [4]. The complex relationship between body mass and electron pathways through erythrocyte plasma membrane has already been addressed [33]. Endothelial cells also possess TPMET systems that transfer intracellular reducing equivalents to blood-borne electron acceptor and whose activity is modulated by cytoplasmic redox status [34] as well as by extracellular molecules such as homocysteine [35]. In endothelium-denuded bovine pulmonary arteries, increasing haeme oxygenase 1 activity is associated with an attenuation of pulmonary artery relaxation and soluble guanylate cyclase activation responses to nitric oxide [36]. Increased haeme oxygenase 1 activity could attenuate relaxation to nitric oxide through depleting the haeme of soluble guanylate cyclase, which controls its cyclic guanosine monophosphate generating activity. Since TPMET activity is highly correlated with the intracellular balance of the NADH/NAD redox pair [34] and with the expression of haeme oxygenase 1 [5], the vasoactive actions of this enzymatic activity may partly contribute to the observed relation between BP and RBC vfcy.

AASI, recently suggested as a marker of arterial stiffness, resulted to be significantly heritable both before and after adjustment for classical covariates [37]. In our (albeit small) sample of non-diabetic people, on the contrary, AASI was correlated with a metabolic variable such as plasma glycaemia. However, it should be noticed that our sample is older than Swedish healthy siblings. Evidence on this regard suggest that: 1) in diabetic rats, elastic properties of the aorta are impaired, being closely related to hyperglycemia-induced vascular wall remodelling [38], 2) in a substudy of the Finnish population-based Health 2000 Survey, FPG levels were independently associated with increased arterial stiffness [39], 3) over a 3-year period in 2080 Japanese men BP and FPG levels even below those defining hypertension and diabetes mellitus, respectively, could synergistically lead to progression of arterial stiffening [40].

In conclusion, non-diabetic siblings of T1D patients show signs of reduced insulin sensitivity, larger circadian amplitude of SBP and higher ambulatory arterial stiffness index. Their broader range of BMI and FPG (increasing from low-normal FPG to IFG) portends nascent or established glucoregulatory perturbations and is associated with consensual perturbations in vascular homeostasis. Moreover, our findings support the proposed role of TPMET systems in vascular pathobiology, which deserves further clinical investigations.

## Limitations

We are aware that repeated ABPM recordings could be recommended to correctly estimate the consistency of BP abnormalities and diagnose circadian hyper-amplitude-tension [26]. However, the use of a single ABPM is common in literature also in consideration of the cost/benefit ratio. General recommendations (2007 ESH-ESC arterial hypertension guidelines) suggest the use of the ABPM when clinic BP shows unusual variability and when symptoms suggest the possibility of hypertension, which is not the case for our siblings and controls at baseline. Obtaining another ABPM is advisable if the first examination has less than 70% of the expected number of valid values because of artefacts.

## Abbreviations

AASI: ambulatory arterial stiffness index; ABPM: ambulatory blood pressure monitoring; ACR: albumin creatinine ratio; BMI: body mass index; DBP: diastolic blood pressure; FPG: fasting plasma glucose; FPI: fasting plasma insulin; HOMA-IS: homeostasis model assessment of insulin sensitivity; HRV: heart rate variability; MESOR: midline-estimating statistic of rhythm; PP: pulse pressure; RBC vfcy: erythrocyte velocity of ferricyanide reduction; SBP: systolic blood pressure; T1D: type 1 diabetes; TPMET: transplasma membrane electron transport

## Competing interests

The authors declare that they have no competing interests.

## Authors' contributions

EM and OG participated in the design, interpretation of the study, analysis of the data, and review of the manuscript. CC and MCM participated in the methodology of the study. All authors read and approved the final manuscript.
